# Immune phenotype of patients with stage IV metastatic inflammatory breast cancer

**DOI:** 10.1186/s13058-020-01371-x

**Published:** 2020-12-02

**Authors:** Sandra V. Fernandez, Alexander W. MacFarlane, Mowafaq Jillab, Maria F. Arisi, Jennifer Yearley, Lakshmanan Annamalai, Yulan Gong, Kathy Q. Cai, R. Katherine Alpaugh, Massimo Cristofanilli, Kerry S. Campbell

**Affiliations:** 1 Department of Medical Oncology, Fox Chase Cancer Center, Philadelphia, PA 19111 USA; 2 Blood Cell Development and Function Program, Institute for Cancer Research, Fox Chase Cancer Center, 333 Cottman Ave, Philadelphia, PA 19111 USA; 3 Present address: Thomas Jefferson University, Sidney Kimmel Medical School, Philadelphia, PA 19107 USA; 4 Merck & Co, Inc., Kenilworth, NJ 07033 USA; 5 Department of Pathology, Fox Chase Cancer Center, Philadelphia, PA 19111 USA; 6 Histopathology Facility, Fox Chase Cancer Center, Philadelphia, PA 19111 USA; 7 Protocol Support Laboratory, Institute for Cancer Research, Fox Chase Cancer Center, Philadelphia, PA 19111 USA; 8 Present address: Northwestern University, Feinberg School of Medicine, Chicago, IL 60611 USA

**Keywords:** Inflammatory breast cancer (IBC), Stage IV IBC, Metastatic IBC, Lymphopenia, PD-1, PD-L1, Immunotherapy, Checkpoint inhibitors, T cells, NK cells, Tumor-infiltrating lymphocytes, Tumor microenvironment

## Abstract

**Background:**

Inflammatory breast cancer (IBC) is a rare but aggressive carcinoma characterized by severe erythema and edema of the breast, with many patients presenting in advanced metastatic disease. The “inflammatory” nature is not due to classic immune-mediated inflammation, but instead results from tumor-mediated blockage of dermal lymphatic ducts. Previous work has shown that expression of PD-L1 on tumor cells can suppress T cell activation in triple-negative (TN) non-IBC breast cancer. In the present work, we investigated immune parameters in peripheral blood of metastatic IBC patients to determine whether cellular components of the immune system are altered, thereby contributing to pathogenesis of the disease. These immune parameters were also compared to PD-1 and PD-L1 expression in IBC tumor biopsies.

**Methods:**

Flow cytometry-based immune phenotyping was performed using fresh peripheral blood from 14 stage IV IBC patients and compared to 11 healthy age-similar control women. Immunohistochemistry for CD20, CD3, PD-1, and PD-L1 was performed on tumor biopsies of these metastatic IBC patients.

**Results:**

IBC patients with Stage IV disease had lymphopenia with significant reductions in circulating T, B, and NK cells. Reductions were observed in all subsets of CD4^+^ T cells, whereas reductions in CD8^+^ T cells were more concentrated in memory subsets. Immature cytokine-producing CD56^bright^ NK cells expressed higher levels of FcγRIIIa and cytolytic granule components, suggesting accelerated maturation to cytolytic CD56^dim^ cells. Immunohistochemical analysis of tumor biopsies demonstrated moderate to high expression of PD-1 in 18.2% of patients and of PD-L1 in 36.4% of patients. Interestingly, a positive correlation was observed between co-expression levels of PD-L1 and PD-1 in tumor biopsies, and higher expression of PD-L1 in tumor biopsies correlated with higher expression of cytolytic granule components in blood CD4^+^ T cells and CD56^dim^ NK cells, and higher numbers of CD8^+^ effector memory T cells in peripheral blood. PD-1 expression in tumor also correlated with increased infiltration of CD20^+^ B cells in the tumor.

**Conclusions:**

Our results suggest that while lymphocyte populations are severely compromised in stage IV IBC patients, an immune response toward the tumor had occurred in some patients, providing biological rationale to evaluate PD-1/PD-L1 immunotherapies for IBC.

**Supplementary information:**

The online version contains supplementary material available at 10.1186/s13058-020-01371-x.

## Background

Inflammatory breast cancer (IBC) is a rare and aggressive malignancy. In the United States (US), IBC accounts for 2–6% of all patients with breast cancer [1, 2]. Although IBC is a relatively rare clinical subtype of locally advanced breast cancer, it is responsible for approximately 10% of breast cancer-associated deaths annually in the US, which translates into about 4000 deaths per year [3, 4]. Approximately 20–30% of IBC patients present with distant metastasis (stage IV disease) at diagnosis, compared to 6–10% of patients with breast cancer that is not inflammatory (non-IBC) [5]. Although recent trends indicate an improvement in survival in IBC patients, the prognosis remains worse than non-IBC cases. The median overall survival (OS) for patients with stage III IBC is 4.75 years, compared to 13.40 years in those with non-IBC, and for stage IV disease OS is 2.27 years in IBC patients versus 3.40 years in non-IBC patients [6, 7]. IBC patients tend to be younger compared to other breast cancer patients, with a median age at diagnosis of 52 years compared to 57 for non-IBC patients [8].

The principal clinical symptoms of IBC are breast erythema, edema, peau d’orange, and dermal lymphatic invasion [9]. Despite its name, IBC is not associated with a profuse inflammatory response. Rather, the characteristic redness and swelling of the breast are due to obstruction of lymphatic channels in the dermis by tumor cells [4, 10].

The current consensus regarding clinical management of IBC includes neoadjuvant systemic therapy (chemotherapy or chemotherapy plus targeted therapy), modified radical mastectomy and level I and II ipsilateral axillary node dissection, post-mastectomy radiotherapy of the chest wall and nodal basin, and adjuvant targeted therapy and hormonal therapy [11]. In the case of stage IV de novo IBC, primary systemic therapy is also recommended, but the decision to use surgery and radiation therapy should be evaluated using a multidisciplinary approach, particularly in those patients who have significant clinical response to systemic therapy [11]. Taxane, doxorubicin, and cyclophosphamide in neoadjuvant systemic therapies have been recommended for stage III primary breast cancer, but there is a clear lack of clinical trials specifically for IBC [11]. For HER2^+^ disease, dual anti-HER2 therapy (pertuzumab and trastuzumab) combined with chemotherapy is recommended [11].

Data on risk factors for IBC are limited, and the contributions of hereditary versus environmental or life-style factors remain poorly understood [12]. Although IBC, like non-IBC breast cancers, is a heterogeneous disease and can occur as any of the five molecular subtypes, the disease is most commonly either HER2 overexpressing or triple-negative (TN) [13]. TN breast cancer, which is defined by absence of estrogen and progesterone receptors, and a lack of HER2 overexpression, has a poorer prognosis than other subtypes [14]. Importantly, the TN phenotype breast cancer (non-IBC) has been associated with higher expression of PD-L1 on tumor cells [15]. The interaction of the PD-1 receptor on T cells with its ligand, PD-L1 on tumor- and immune-infiltrating cells, suppresses T cell-mediated immune responses and may play a role in immune escape by human tumors [16]. The extensive accumulation of tumor emboli in the lymphatic vessels of IBC patients supports the notion that the host immune surveillance system is suboptimal or that the tumor cells have adopted immune escape mechanisms to avoid detection by the host. Several recent studies have provided evidence for immune responses toward IBC, suggesting that patients may benefit from immunotherapies, such as PD-1/PD-L1 blocking antibodies [17–22]. Nonetheless, there is insufficient information on peripheral blood leukocyte immune-phenotypes in IBC patients. In the present work, we utilized flow cytometry and immunohistochemistry analysis to investigate immune parameters of metastatic IBC patients and compared them to healthy female volunteer donors. In addition, we studied the expression of PD-L1 and PD-1 in tumor biopsies of these patients with metastatic IBC. Our study aims to determine whether cellular components of the immune system are altered in IBC patients, thereby contributing to the pathogenesis of the disease.

## Methods

### Human subjects and blood sample preparation

The blood samples used in this study were collected from 14 IBC patients with stage IV disease, who were treated at Fox Chase Cancer Center (FCCC) between 2010 and 2012. Control blood samples were collected and anonymized through the FCCC Biosample Repository from 11 age-similar female healthy volunteers. This study was approved by both the research review committee (RRC) and the institutional review board (IRB) at FCCC. All patients and healthy controls signed IRB-approved informed consent and HIPAA certification prior to sample collection. Retrospective chart reviews were performed in order to collect patient data, which included age at diagnosis, hormone receptor subtype, and treatment history, as summarized in Table 1. Peripheral blood was collected from IBC patients prior to starting a new line of chemotherapy and was processed within 6 h of collection. The blood was used to determine total and differential counts of leukocytes and to identify frequencies of lymphocyte subpopulations (T, B, and NK cells) according to standardized protocols of immune phenotyping by flow cytometry, as previously described [23, 24]. Whole blood (20 ml) was drawn into heparinized tubes and subsequently mixed in equal proportions with complete RPMI 1640 medium (supplemented with 10% fetal bovine serum, 100 μg/ml penicillin/streptomycin, 2 mM L-glutamine, 10 mM HEPES, 1 mM sodium pyruvate, and 50 μM 2-mercaptoethanol), layered over Lymphoprep (Axix-shield POC AS, Oslo, Norway), and centrifuged at 500*g* for 30 min. The buffy coat was removed and suspended with staining buffer (RPMI1640 without biotin or phenol red, and supplemented with 2.0 g/L NaHCO_3_ and 2.4 g/L HEPES, pH 7.0).

**Table 1:** Demographics of stage IV IBC patients

Patient ID	Age at diagnosis	Race	IBC Subtype at diagnosis	Months after diagnosis of blood collection and treatment status	Overall Survival (OS)
Patient 1	42	H	ER^−^PR^−^Her2^+^	Month 18 Treated	67 months
Patient 2	47	C	TN Secondary IBC	Month 12 Treated	27 months
Patient 3	55	C	TN	Month 20 Treated	38 months
Patient 5	49	C	ER^−^PR^−^Her2^+^	Month 30 Treated	44 months*
Patient 6	66	C (J)	ER^+^PR^−^Her2^−^ (became TN at month 8)	Month 8 Treated	10 months (cardiac disease)
Patient 7	48	C	TN	Month 21 Treated	27 months
Patient 8	32	C	ER^+^PR^−^Her2^−^	Month 50 Treated	105 months
Patient 9	55	A	ER^−^PR^−^Her2^+^	Month 4 Treatment naive	16 months
Patient 10	62	C	TN	Month 32 Treated	37 months
Patient 11	61	C	TN	Month 13 Treated	31 months
Patient 13	43	C	ER^+^PR^+^Her2^+^	Month 1 Treatment naive	87 months
Patient 14^†^	44	C	ER^−^PR^−^Her2^+^	Month 13 Treated	115 months
Patient 15	30	C	ER^+^PR^−^Her2^+^	Month 22 Treated	39 months
Patient 16	69	A	ER^−^PR^−^Her2^+^	Month 26 Treated	37 months

Age at disease onset, race, IBC subtype, time in which blood samples were collected since disease onset, and survival is indicated for each patient. All the patients had metastatic disease at the time of the blood collection and received multiple therapies with the exception of patients 9 and 13

*C* Caucasian, *TN* triple-negative, *A* Asian, *H* Hispanic, *J* Jewish heritage

*Last record available

^†^Patient was breast feeding her baby when she was diagnosed with IBC

### Antibodies and cell staining for flow cytometry

The staining panel, monoclonal antibody clones, and sources are shown in Supplementary Table S1. Antibodies in direct surface staining tubes were directly conjugated with fluorophores. Staining for perforin and granzyme B was done after samples were fixed with 2% paraformaldehyde and permeabilized with PBS containing 0.1% saponin, 1% BSA, and 0.1% sodium azide. Staining for FoxP3 was performed after cells were fixed and permeabilized with the Biolegend FOXP3 Fix/Perm Buffer Set (#421403). One million cells were stained in each sample on ice for 20 min in approximately 200 μl of staining buffer and rinsed twice. Staining tubes that were not fixed/permeabilized were subjected to 100 ng/ml propidium iodide (Invitrogen) in the second rinsing step to mark dead cells. The BD IMK kit (Catalog # 340503) was used to determine the percentages and absolute counts in whole blood of the following mature lymphocytes: T lymphocytes (CD3^+^), B lymphocytes (CD19^+^), helper/inducer T lymphocytes (CD3^+^CD4^+^), cytotoxic T lymphocytes (CD3^+^CD8^+^), and natural killer (NK) lymphocytes (CD3^−^CD16^+^ and/or CD3^−^CD56^+^). BD Trucount™ tubes were used for determining absolute counts.

### Flow cytometry instrumentation and data analysis

Stained cells were analyzed on a Beckman Dickinson (BD) ARIA II flow cytometer with 4 lasers at 633 nm, 488 nm, 405 nm, and 365 nm wavelengths. Absolute lymphocyte counts were analyzed on a BD FACS Calibur flow cytometer. Data were collected with BD FACS Diva software version 6 and analyzed with Flowjo v9.2 (Tree Star Inc., Ashland, OR), Microsoft Excel (v12), GraphPad Prism v5.0d or later (GraphPad Software Inc., La Jolla, CA), and Matlab R2016b (The Mathworks). Single cell events were first gated by a forward scatter height vs. forward scatter area plot and viable cells were then gated by lack of propidium iodide staining. Viable CD45^+^ cells were split into myeloid and lymphocyte populations by applying a side scatter gate and then divided into sub-populations based on the expression of CD3, CD14, CD16, CD19, CD20, CD27, CD62L, CD45RA, and CD56. Regulatory T (Treg) cells were quantified as a percent of CD4^+^ T cells that were CD25^high^ and FOXP3^+^. Immune parameters measured are shown in Supplementary Table S2.

### Analysis of PD-1 and PD-L1 expression in tumor biopsies

Surgically obtained tumor samples were placed in 10% formalin buffer, processed and embedded in paraffin (FFPE), and underwent pathological examination for diagnosis. Whole tissue sections cut from FFPE tissue blocks were deparaffinized and rehydrated with serial passage through changes of xylene and graded ethanol. All slides were subjected to heat-induced epitope retrieval in Envision FLEX Target Retrieval Solution, High pH (Dako, Carpinteria, CA). Endogenous peroxidase in tissues was blocked by incubation of slides in 3% hydrogen peroxide solution prior to incubation with primary antibody (anti-PD-L1, clone 22C3, Merck & Co. Inc., Palo Alto CA, USA, or anti-PD-1 clone NAT105, Cell Marque, Rocklin, CA, for 60 min). Antigen-antibody binding was visualized via application of the FLEX+ polymer system (Dako, Carpinteria, CA) for PD-1 and PD-L1, and application of 3, 3′ diaminobenzidine (DAB) chromogen (Dako, Carpinteria, CA). Stained slides were counterstained with hematoxylin and cover slipped for review. Stained sections then underwent semi-quantitative evaluation of positive cell frequency (0 = none, 1 = rare, 2 = low, 3 = moderate, 4 = high, 5 = very high), as previously described [25]. Scoring was conducted by a pathologist, with scores incorporating prevalence of both tumor cells and non-tumor cell labeling. PD-1 and PD-L1 expression scores of > 2 were considered positive in combined tumor and non-tumor cells on the 0–5 scale.

### Measuring tumor-infiltrating lymphocytes (TIL)

Immunohistochemistry was used to differentiate TIL in the FFPE tumor samples, by staining for CD3 (T cells) and CD20 (B cells). The antibodies and staining procedures used were anti-CD3 prediluted (clone 2GV6; Ventana/Roche, US) and incubated 32 min at 37 °C; anti-CD20 (clone L26; Dako) prediluted (1:1280 in Ventana antibody dilution buffer), incubation 32 min at 37 °C. The specimens were then counterstained with hematoxylin. For each analysis, evaluation was conducted in the tumor and immediately peri-tumoral areas with quantitation output as the percentage of positive cells relative to total nucleated cells.

### Statistical methods

Flow cytometry data for distinct parameters were quantified either as geometric mean fluorescence intensity (GMFI) or percentage of cells that express a cell surface receptor. Comparisons between immune cell parameters from healthy donors and IBC stage IV patients were performed with a Wilcoxon rank-sum test. *P* and *R* values for the significance of correlations between immune parameters were determined using a Spearman test. False discovery rate (FDR) analysis was further performed for comparisons within flow cytometry data using the Benjamini-Hochberg method and significance was defined as an FDR of < 20% [26].

## Results

### Population studied

Fourteen female patients with metastatic IBC (stage IV) and 11 healthy female age-similar volunteers were included in the study (characteristics summarized in Table 1). The median age of the IBC patient population at disease onset was 49.5 years old (range 30–69 years old) and the median age at the time of blood drawn was 50.5 years old (range 31–72). The median age of healthy donors was 54 (range 34–70). From the 14 metastatic IBC patients, 2 patients were treatment naïve at the time of blood collection (patients 9 and 13 in Table 1). Of the 12 patients that were treated, 5 patients had ER^−^PR^−^Her2^−^ (triple-negative, TN) IBC subtype; 4 patients had ER^−^PR^−^Her2^+^ IBC; 1 patient had ER^+^PR^−^Her2^+^ IBC; and 2 patients had ER^+^PR^−^Her2^−^ IBC at the time of blood collection (Table 1). From patients without treatment at the time of enrollment in the study, 1 patient had ER^−^PR^−^Her2^+^ disease and another had ER^+^PR^+^Her2^+^ IBC. The median overall survival for the 5 TN and 9 non-TN patients was 31 months and 44 months, respectively (Table 1).

### Lymphocytes in metastatic IBC

**Fig. 1:**
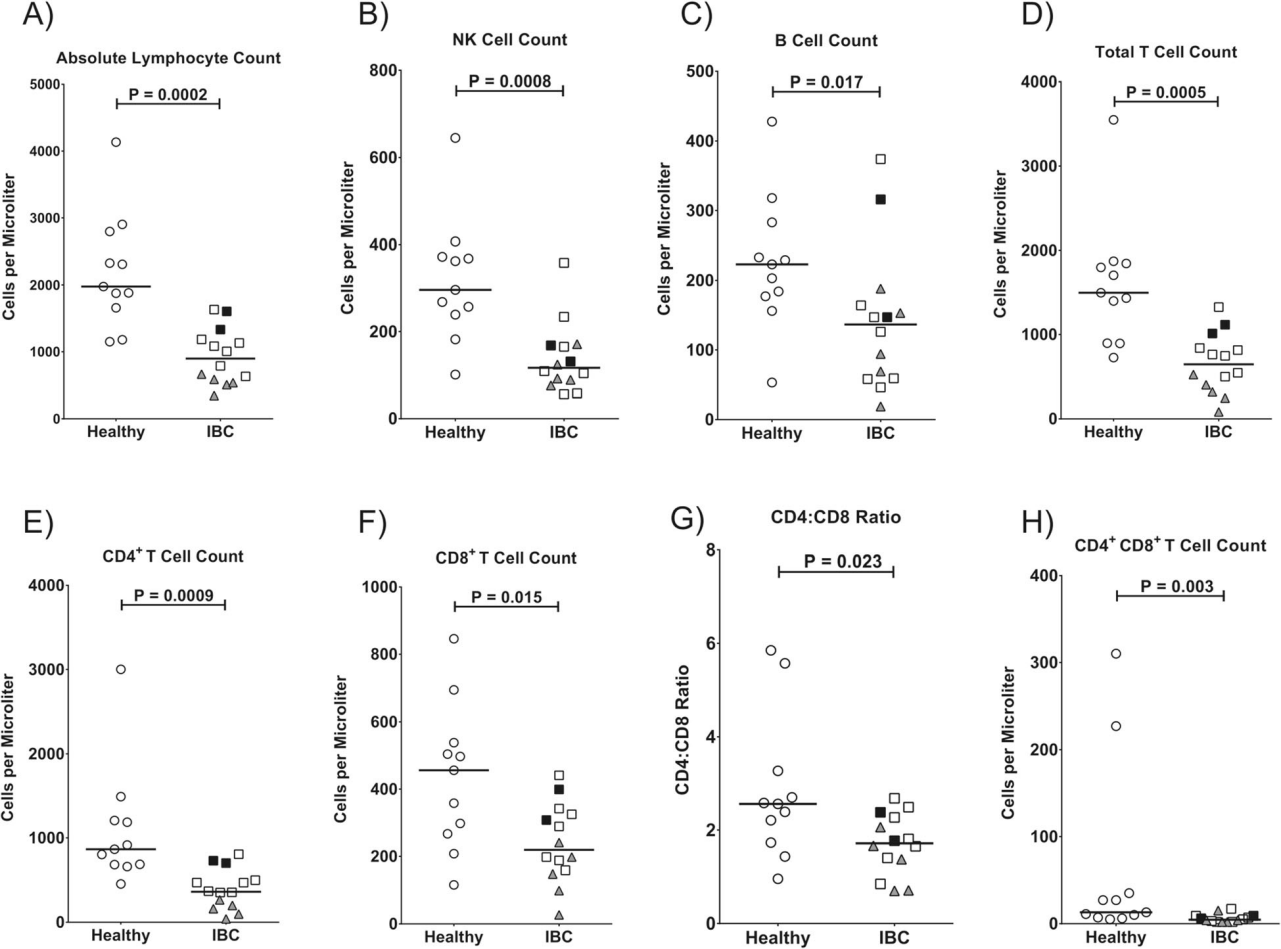
Frequencies of major lymphocyte types in peripheral blood of IBC patients compared to healthy controls. Absolute counts per μl of peripheral blood were determined for **a** total lymphocytes, **b** CD3^−^CD56^+^ NK cells, **c** CD19^+^ B cells, **d** CD3^+^ T cells, **e** CD3^+^CD4^+^ T cells, **f** CD3^+^CD8^+^ T cells, **g** CD4^+^ to CD8^+^ ratio of CD3^+^ T cells, and **h** CD3^+^CD4^+^CD8^+^ T cells. Healthy donors are shown as open circles, triple-negative IBC patients as shaded triangles, untreated IBC patients as filled squares, and chemotherapy-treated IBC patients as open squares. Horizontal lines indicate median values and statistical significance was determined by a Wilcoxon rank-sum test

**Fig. 2:**
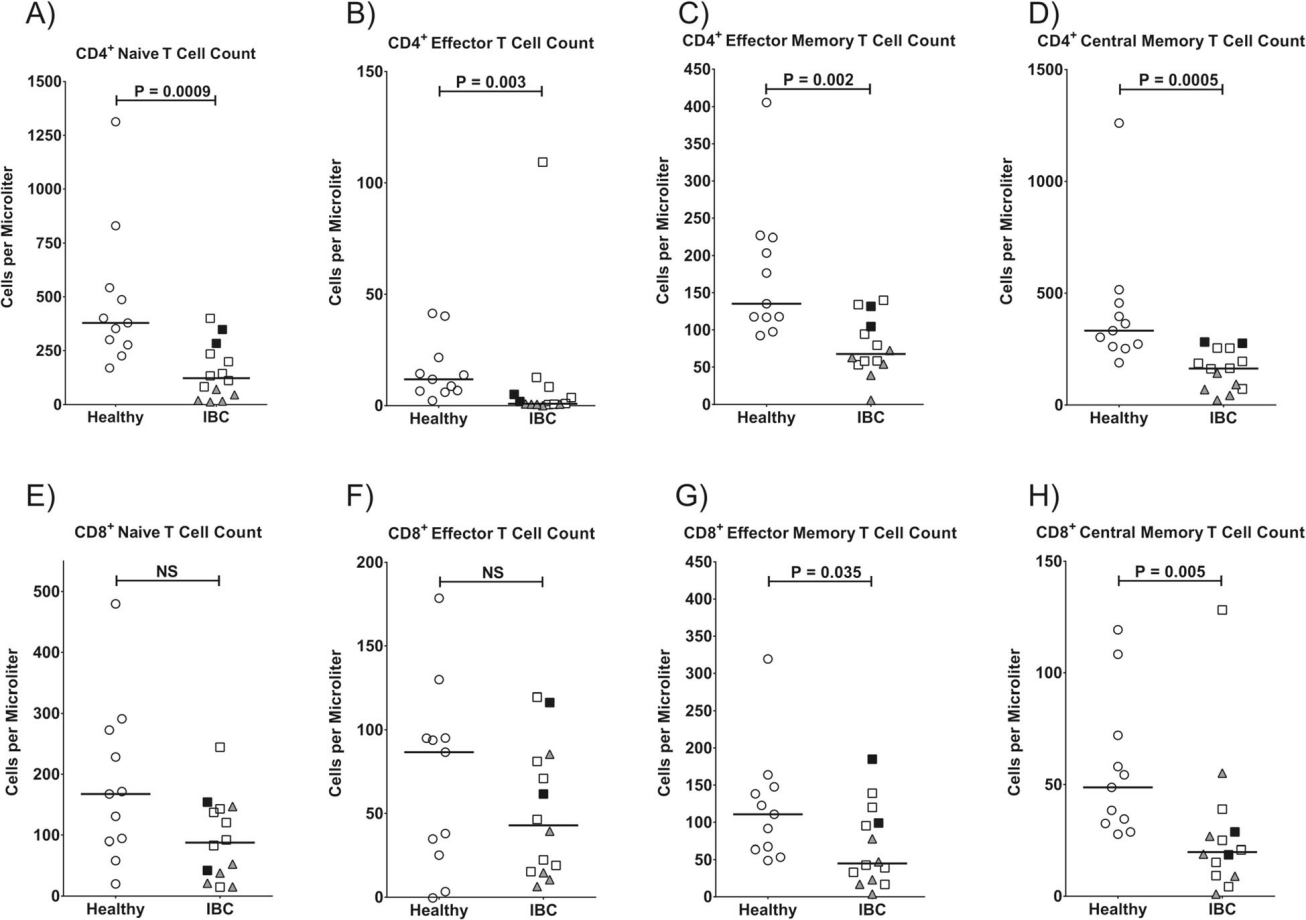
Frequencies of T cell subsets in peripheral blood of IBC patients compared to healthy controls. Absolute counts per μl of peripheral blood were determined for **a** CD4^+^ naive (CD62L^+^ CD45RA^+^) T cells, **b** CD4^+^ effector (CD62L^−^ CD45RA^+^) T cells, **c** CD4^+^ effector memory (CD62L^−^ CD45RA^−^) T cells, **d** CD4^+^ central memory (CD62L^+^ CD45RA^-^) T cells, **e** CD8^+^ naive T cells, **f** CD8^+^ effector T cells, **g** CD8^+^ effector memory T cells, and **h** CD8^+^ central memory T cells. Healthy donors are shown as open circles, triple-negative IBC patients as shaded triangles, untreated IBC patients as filled squares, and chemotherapy-treated IBC patients as open squares. Horizontal lines indicate median values and statistical significance was determined by a Wilcoxon rank-sum test

**Fig. 3:**
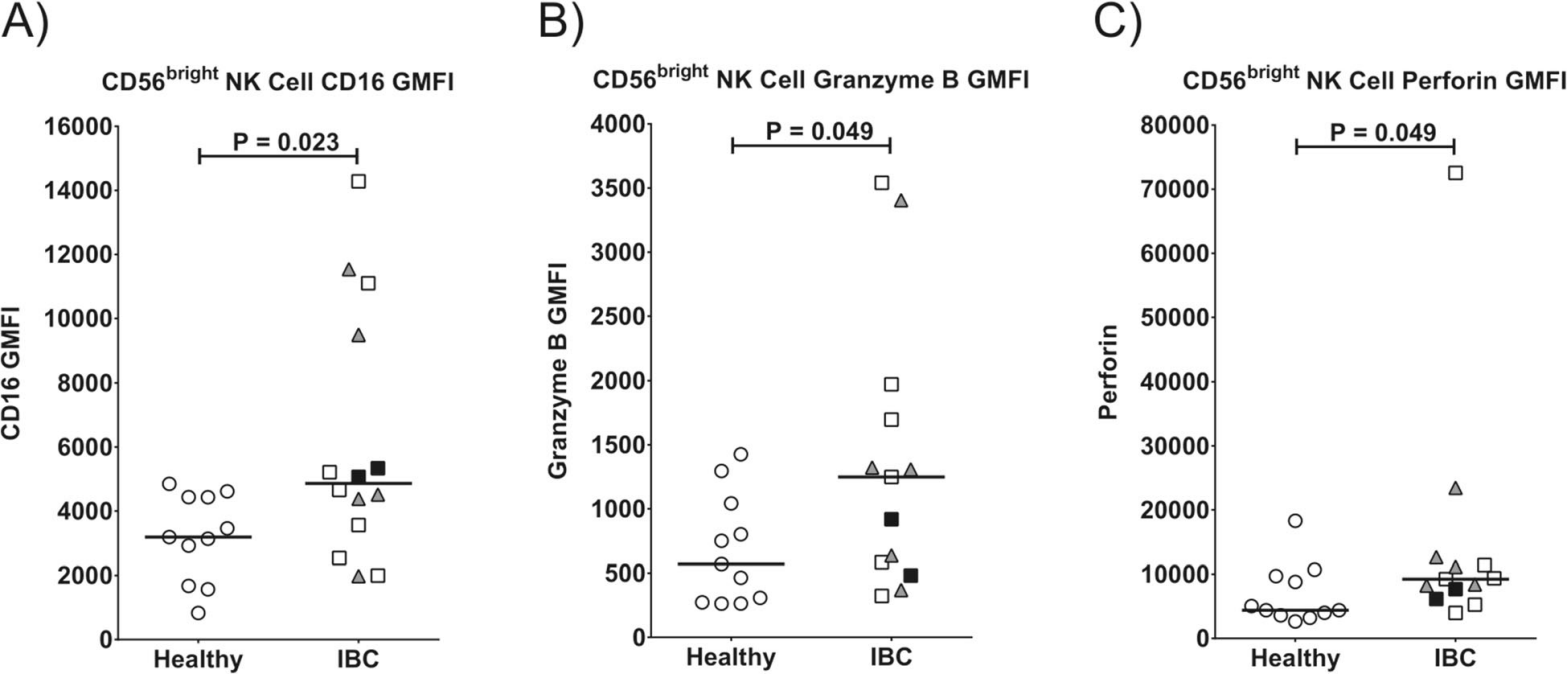
Expression levels of CD16, granzyme B, and perforin in NK cell subsets from IBC patients compared to healthy controls. Expression levels CD56^bright^ NK cells as measured by geometric mean fluorescence intensity (GMFI) for expression levels of **a** CD16 (FcγRIIIA), **b** granzyme B, and **c** perforin. Healthy donors are shown as open circles, triple-negative IBC patients as shaded triangles, untreated IBC patients as filled squares, and chemotherapy-treated IBC patients as open squares. Horizontal lines indicate median values and statistical significance was determined by a Wilcoxon rank-sum test

We performed comprehensive immune phenotyping by multi-parameter flow cytometry on fresh peripheral blood samples from each of the IBC patients and healthy controls. Seventy-two immune parameters (Supplementary Table S2) were compared between IBC and healthy donors by Wilcoxon rank-sum test. The absolute counts of T cells (CD3^+^), B cells (CD19^+^ CD3^−^), helper T cells (CD3^+^ CD4^+^), cytotoxic T cells (CD3^+^ CD8^+^), and natural killer (NK) cells (CD3^−^ CD56^+^) in blood were also determined, and median values are shown in Table 2. IBC patients with metastatic disease had a significantly lower absolute lymphocyte count than healthy female donors (median 897 ± 413 versus 1976 ± 855; Fig. 1a). The absolute number of total NK cells in the peripheral blood of IBC patients was also lower than in healthy donors (117 ± 80 versus 296 ± 142; Fig. 1b), which paralleled reductions in absolute numbers of B cells (137 ± 101 versus 223 ± 96; Fig. 1c) and T cells (646 ± 350 versus 1496 ± 763; Fig. 1d). The five IBC patients with TN disease exhibited some of the most extreme lymphopenia with particularly low levels of T cells (Fig. 1a, d; TN IBC patients are shown as shaded triangles in Figs. 1, 2, and 3). It is also worth noting that lymphocyte counts from the two patients that did not receive chemotherapy treatment were similar to other IBC patients (especially comparable to the non-TN treated patients; shown as filled squares in Figs. 1, 2, and 3).

**Table 2:** The median number ± standard deviation of lymphocytes per μl is indicated for 14 stage IV metastatic IBC patients and 11 healthy donors as assessed using BD Trucount™ assays

	IBC patients	Healthy donors	***p*** values
Total lymphocytes per μl	897 ± 413	1976 ± 855	0.00018
CD3^+^ T cells per μl	646 ± 350	1496 ± 762	0.00051
CD8^+^ T cells per μl	222 ± 117	458 ± 215	0.015
CD4^+^ T cells per μl	372 ± 236	873 ± 701	0.00093
CD4^+^CD8^+^ cells per μl	6 ± 5	14 ± 104	0.0030
NK cells per μl	117 ± 80	296 ± 142	0.00076
B cells per μl	137 ± 101	223 ± 96	0.017
CD4/CD8 ratio	1.7 ± 0.7	2.6 ± 1.6	0.023
CD4^+^ central memory cells per μl	165 ± 88	335 ± 296	0.00051
CD4^+^ effector cells per μl	1 ± 29	12 ± 13	0.0034
CD4^+^ effector memory cells per μl	68 ± 39	136 ± 91	0.0016
CD4^+^ naïve cells per μl	125 ± 126	382 ± 330	0.00093
CD8^+^ central memory cells per μl	20 ± 32	49 ± 32	0.0048
CD8^+^ effector cells per μl	43 ± 39	87 ± 56	0.37
CD8^+^ effector memory cells per μl	46 ± 54	112 ± 77	0.035
CD8^+^ naïve cells per μl	89 ± 68	168 ± 131	0.052
CD16 GMFI on CD56^bright^ NK cells	4896 ± 3883	3224 ± 1361	0.023
Granzyme B GMFI on CD56^bright^ NK cells	1257 ± 1064	578 ± 474	0.049
Perforin GMFI on CD56^bright^ NK cells	9412 ± 18,059	4564 ± 4717	0.049

All comparisons with significant *p* values < 0.05 also passed false discovery rate testing

### Different subpopulations of T lymphocytes

The absolute number of CD4^+^ T helper lymphocytes in the peripheral blood of metastatic IBC patients was significantly lower than in healthy donors (372 ± 236 versus 873 ± 701, respectively; Fig. 1e), with the lowest numbers in the TN patients. Significant reductions were also noted in the absolute numbers of CD8^+^ cytotoxic T lymphocytes (222 ± 117 versus 458 ± 215, respectively; Fig. 1f and Table 2). The median CD4/CD8 ratio was also lower at 1.7 ± 0.7 for metastatic IBC patients and 2.6 ± 1.6 for healthy donors (Fig. 1g and Table 2). In addition, the numbers of CD4^+^CD8^+^ T lymphocytes were lower in the IBC patients (6 ± 5 versus 14 ± 104; Fig. 1h and Table 2).

T cell subsets were further delineated by differential staining for CD45RA and CD62L. Lymphopenia was evident in all naive, effector, and memory subsets of CD4^+^ and CD8^+^ T cells, but fell short of statistical significance in the naive and effector CD8^+^ T cell subsets (Fig. 2a–h and Table 2). The more significant reduction of CD4^+^ T cells in TN patients was particularly evident in CD4^+^ naïve and central memory subsets (Fig. 2a, d).

### Natural killer cells

NK cells provide important innate anti-tumor responses in early and metastatic cancers [27]. Most NK cells in the peripheral circulation are highly cytolytic toward certain tumor target cells and express high levels of perforin and granzyme B in cytolytic granules, low levels of the surface marker CD56 (neural cell adhesion molecule 1; CD56^dim^), and high levels of CD16 (FcγRIIIA, low-affinity receptor for the Fc portion of IgG), which can mediate potent antibody-dependent cellular cytotoxicity (ADCC). A less mature, but minor subset of NK cells in human blood expresses high levels of CD56 (CD56^bright^), mediates more cytokine production than cytotoxicity, and generally lacks expression of perforin, granzyme B, and CD16. The amount of CD16 expressed on the surface of immature CD56^bright^ NK cells in IBC stage IV patients was higher than in healthy donors (median 4896 ± 3883 vs. 3224 ± 1361 GMFI; Fig. 3a and Table 2), although the expression was not different in the CD56^dim^ NK cells (data not shown). CD56^bright^ NK cells in metastatic IBC patients also tended to have higher expression of granzyme B (1257 ± 1064 vs. 578 ± 474 GMFI; Fig. 3b and Table 2) and perforin (9412 ± 18,059 vs. 4564 ± 4717 GMFI; Fig. 3c and Table 2) than in healthy donors. All of these changes suggest that the CD56^bright^ NK cells may be expressing markers characteristic of more mature CD56^dim^ cells because they are rapidly maturing to replenish the depleted pool of NK cells.

### PD-1 and PD-L1 expression in IBC tumors

**Fig. 4:**
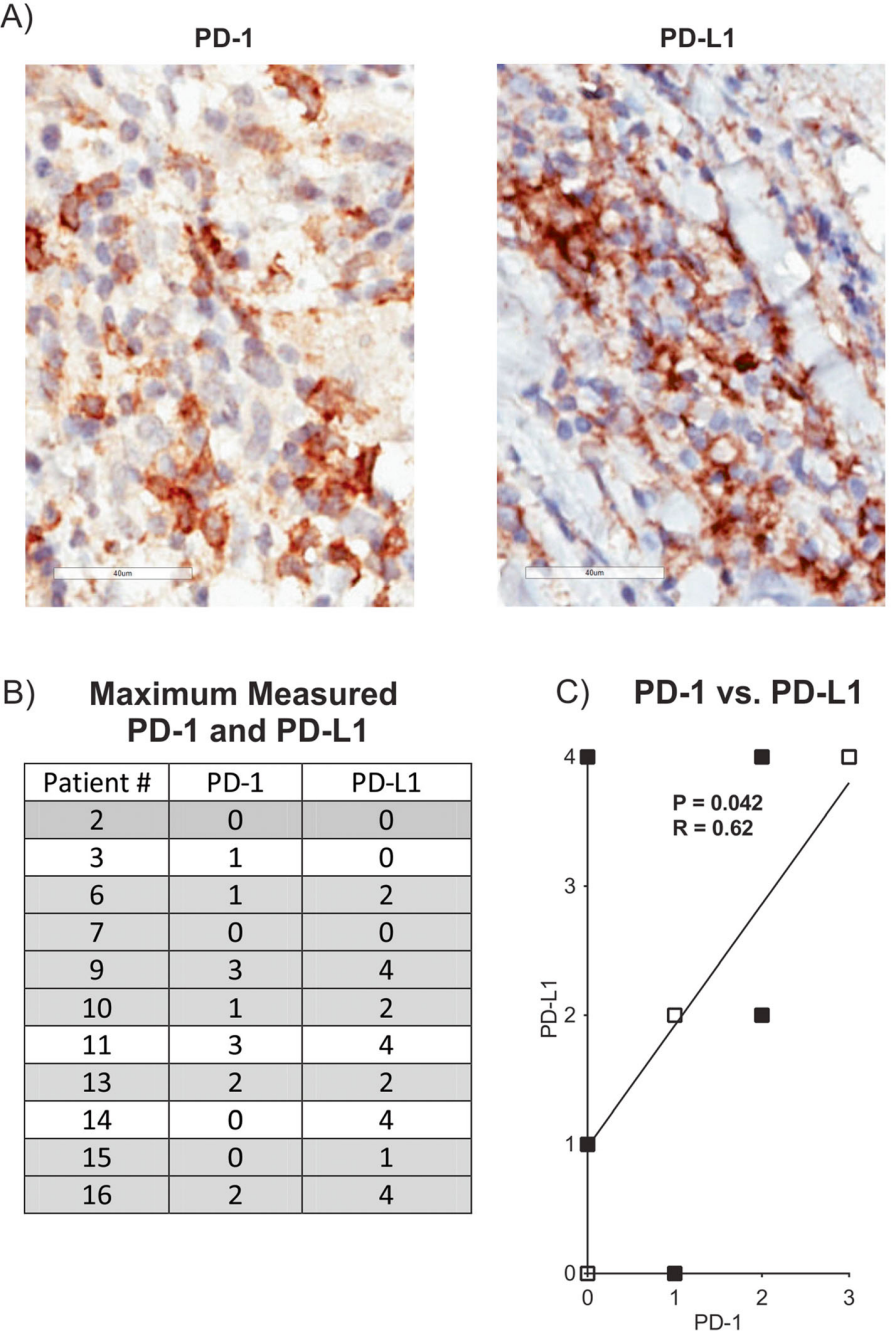
Analysis of PD-1 and PD-L1 expression in tumor biopsies from stage IV IBC patients. Sixteen tumor biopsy samples from 11 patients were stained by immunohistochemistry for PD-1 and PD-L1. Biopsies from patients #1, 5, and 8 were not available for evaluation. Details on biopsies are provided in Supplementary Table S3. **a** PD-1 and PD-L1 staining in brown is shown in samples from a representative patient. Magnification is × 600 in each image and bars designate 40 μm. **b** The expression scores for PD-1 and PD-L1 for tumor biopsy samples from the 11 patients assayed. Score definitions are provided in the “Methods” section. Values represent the maximum measured PD-1 or PD-L1 values in cases where a second measurement was made (all values shown in Supplementary Figure S3). Gray shaded patients had blood samples acquired within 1 month of tumor biopsies and were utilized for blood to tumor comparisons in Fig. 5. **c** Positive correlation between PD-L1 score (*y*-axis) and PD-1 score (*x*-axis) from the 11 patients evaluated using maximum values from panel **b**. Open squares designate overlapping datapoints from two patients. *P* and *R* values were computed from a Spearman test. The line is a least-squares fit to the data that is provided for visual purposes

We were able to evaluate PD-1 and PD-L1 expression levels by immunohistochemistry (IHC) analysis in tumor biopsies from 11 of the 14 stage IV IBC patients. Biopsy details are provided in Supplementary Table S3, and representative IHC staining is shown in Fig. 4a. PD-1 expression with this assay was observed only on TIL, and while PD-L1 expression may be observed on a variety of cell types, it was observed exclusively on infiltrating immune cells in this particular cohort of samples. A 0–5 point system was used to score expression patterns with “5” being the highest level, as described in the “Methods” section. Since more than one tumor sample was evaluated for some patients, we report the highest score from repeat samples as the value for a particular patient in Fig. 4b, but values for all samples are reported in Supplementary Table S3. As shown in Fig. 4c, the highest level of PD-1 expression was scored as “3” (moderate) in only two patients (18.2%). PD-L1 expression levels were more diverse with expression scored as “4” (high) in four patients (36.4%) (Fig. 4b). Interestingly, a positive correlation was observed between co-expression of PD-1 and PD-L1 in the tumor samples from the IBC patients (Fig. 4c).

### Correlations of parameters in blood to PD-L1 in tumor samples

**Fig. 5:**
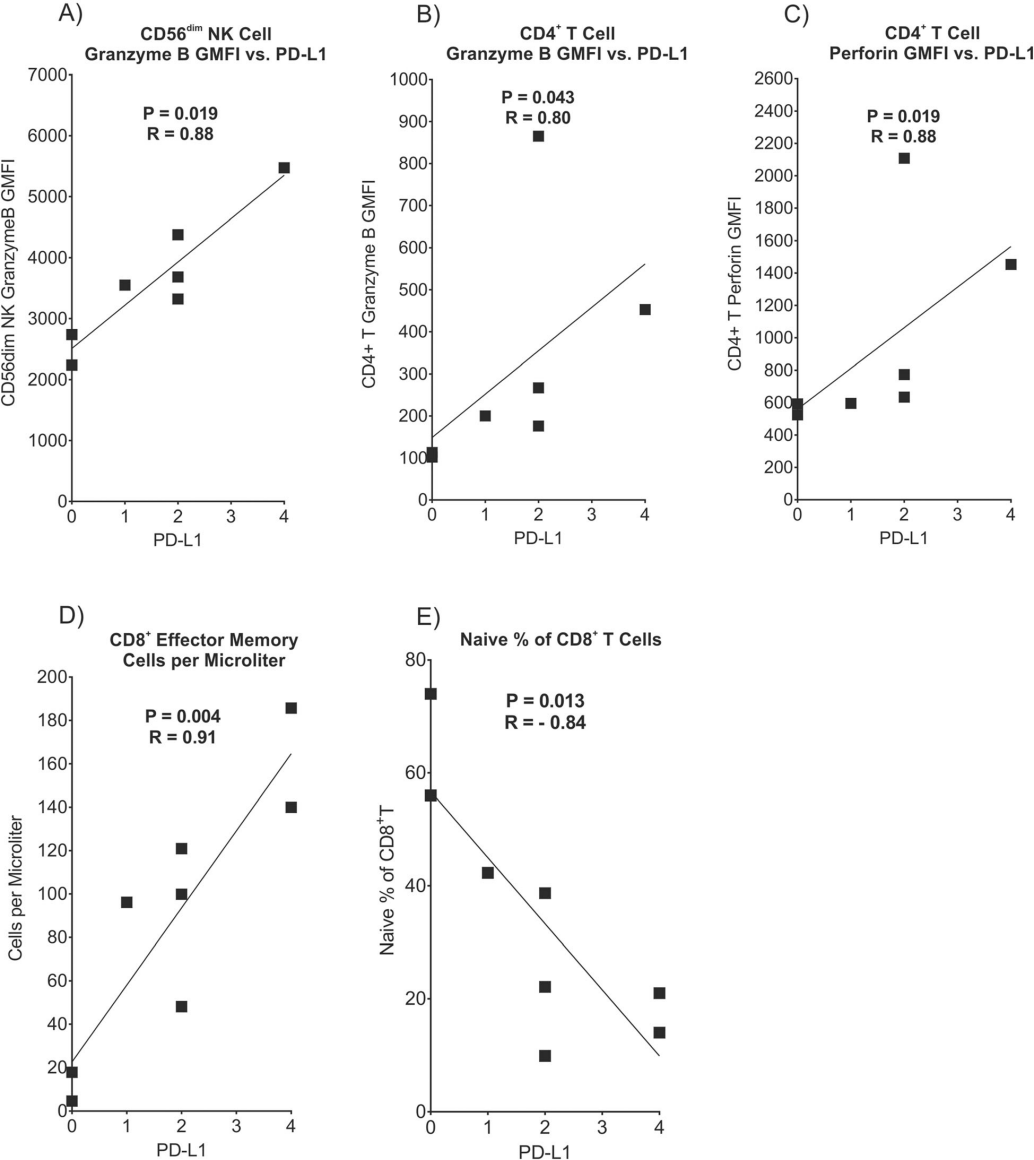
Correlations between PD-L1 expression in tumor samples by IHC and various immune parameters in peripheral blood by flow cytometry. Flow cytometry data from the eight patients that provided blood samples within 1 month of tumor biopsies (Fig. 4b) were correlated with PD-L1 scores in their tumors. Scores for PD-L1 staining from tumor biopsies in individual patients are shown on the *x*-axis in each panel with *y*-axes showing values for significantly correlated flow cytometry parameters from the same patients: **a** Granzyme B GMFI in CD56^dim^ NK cells, **b** granzyme B GMFI in CD4^+^ T cells, **c** perforin GMFI in CD4^+^ T cells, **d** numbers of CD8^+^ effector memory T cells/μl of peripheral blood, **e** % of total CD8^+^ T cells with the naïve phenotype. Datapoints represent the maximum measured PD-L1 values in cases where a second measurement was made, as in Fig. 4. *P* and *R* values were computed from a Spearman test. The lines are least-squares fits to the data that are provided for visual purposes

We next explored whether expression levels of PD-L1 in tumor biopsies correlated with immune biomarkers on T and NK cells in peripheral blood. Eight of the biopsy samples were obtained within 1 month of the blood analyzed by flow cytometry (Fig. 4b and Supplementary Figure S3), and these were used for correlation analysis between tumor and blood samples. A significant positive correlation was observed between PD-L1 in the tumor biopsies and granzyme B expression level in CD56^dim^ NK cells in peripheral blood, as shown in Fig. 5a. A positive correlation was also noted for PD-L1 expression in tumor biopsies and both granzyme B and perforin in CD4^+^ helper T cells in peripheral blood, which characteristically express very low levels of these cytolytic granule-associated proteins (Fig. 5b, c). As shown in Fig. 5d, a higher PD-L1 IHC score in tumor samples was also found to correlate significantly with higher numbers of CD8^+^ effector memory cytotoxic T cells in peripheral blood. A higher PD-L1 score in tumor biopsies was also significantly correlated with a reduced percentage of naïve CD8^+^ T cells in the blood (Fig. 5e). Therefore, our data demonstrate that higher expression of PD-L1 in the stage IV IBC tumor microenvironment (TME) increases cytolytic granule components in blood NK and CD4^+^ T cells and appears to shift the CD8^+^ T cell pool from naïve toward an effector memory phenotype in peripheral blood, suggesting that an immune response had occurred in many of these patients, presumably toward the IBC tumor.

### Correlations of TIL and PD-1 and PD-L1 in tumor samples

**Fig. 6:**
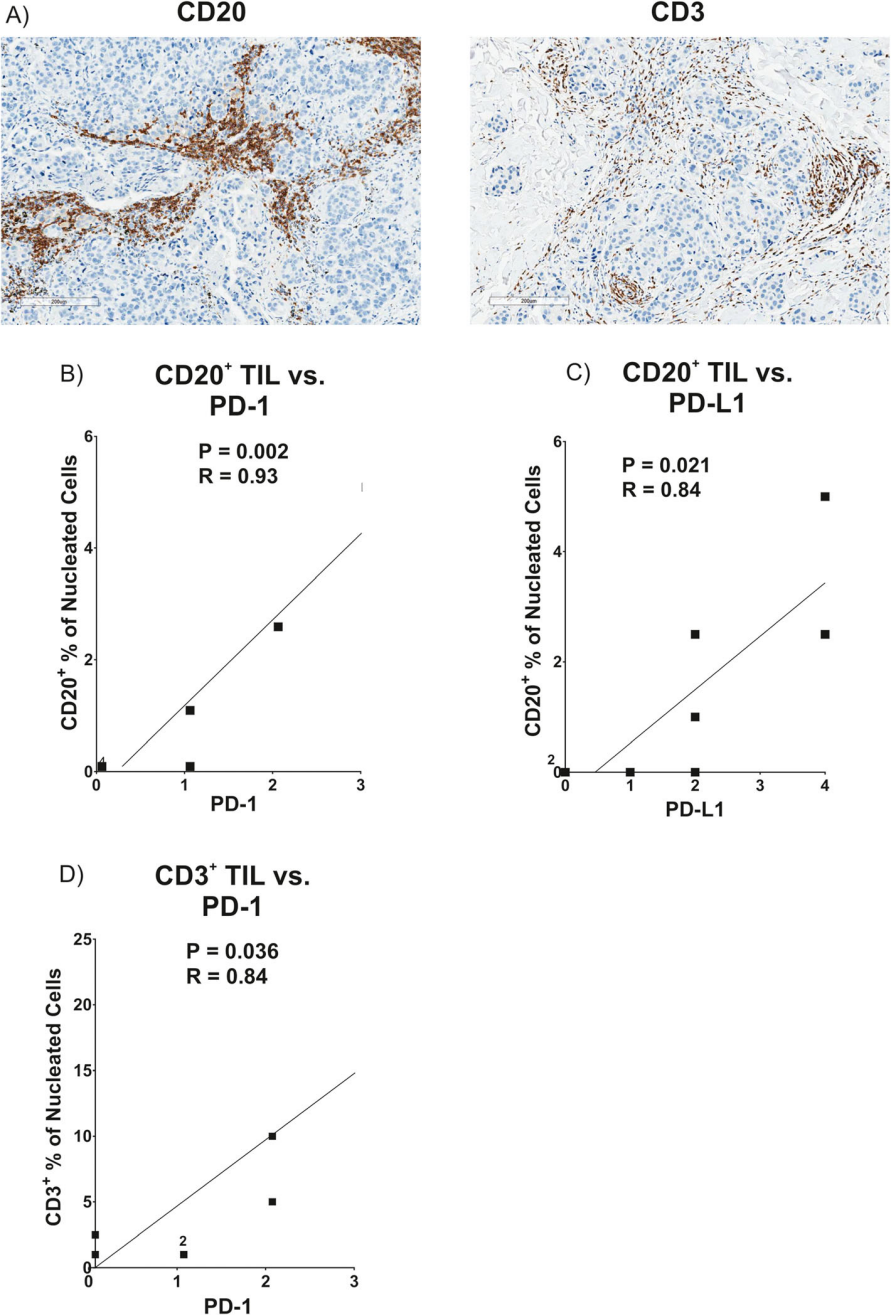
Correlations between PD-1 and PD-L1 expression in tumor samples and infiltrating T and B cells by IHC. The eight tumor samples analyzed in Fig. 5 were stained for CD3 to mark infiltrating T cells, CD20 to mark infiltrating B cells, and hematoxylin. The sample from patient 10 had limited available tissue, so this was excluded from the CD3 staining analysis. **a** CD3 and CD20 staining in brown is shown in samples from a representative patient. Magnification is × 100 in each image and bars designate 200 μm. Percentages of CD3^+^ or CD20^+^ TIL were assessed by a pathologist as percentage of positive cells relative to total nucleated cells in tumor and immediately peri-tumoral areas. Percentage scores for staining of TIL (*x*-axis) were correlated to scores for PD-1 and PD-L1 (*y*-axis) from the same tumor biopsies in individual patients are shown as: **b** % CD20^+^ TIL vs. PD-1, **c** % CD20^+^ TIL vs. PD-L1, and **d** % CD3^+^ TIL vs. PD-1. Numbers next to square icons designate the number of multiple superimposed data points at that position

Given the correlations of PD-L1 expression in the tumors and immune parameters in the blood, we also assessed whether PD-1 and PD-L1 in these tumors correlated with tumor-infiltrating lymphocytes (TIL). The tumors were stained by IHC for CD3 to quantify infiltrating T cells and CD20 to quantify B cells, and examples of staining are shown in Fig. 6a. The infiltrating T and B cells were quantified as percentage of positive cells relative to the nucleated cells within each tumor and immediate peri-tumoral space. The percentage of infiltrating T and B cells did not correlate with any of the immune parameters in the blood. On the other hand, the B cell infiltration correlated significantly with expression of both PD-1 and PD-L1 in tumor, as shown in Fig. 6b, c, although only the correlation with PD-1 passed FDR analysis. A trend was also noted for a correlation between T cell infiltration and expression of PD-1, which was statistically significant by Wilcoxon analysis, but did not pass FDR analysis, as shown in Fig. 6d.

## Discussion

In the present work, we studied the immune profile of 14 IBC patients with stage IV disease and compared to 11 age-similar healthy controls. The analysis of fresh peripheral blood showed that the most notable parameter that differed in the immune cells of the patients with metastatic IBC was lymphopenia. Our data showed significant deficits in numbers of T, NK, and B lymphocytes in peripheral blood of stage IV IBC patients. Although we cannot rule out that some of the lymphopenia in our patient population may have resulted from prior chemotherapy, we show that lymphocyte subset counts from our two treatment naïve IBC patients were generally consistent with treated non-TN IBC patients and nearly always below the medians of our healthy controls, indicating a disease-related impact. A previous study by Reuben and Lee also demonstrated that patients with metastatic IBC had lymphopenia associated with significantly lower CD4^+^ T and B cells, but higher counts of monocytes in peripheral blood as compared to healthy controls, whereas these cell counts were similar to healthy controls in IBC patients with non-metastatic disease [28]. Mego et al. also reported more severe decreases in absolute lymphocyte count of IBC patients with metastatic disease [29].

Our results expand upon previous studies by showing that the numbers of all subpopulations of CD4^+^ helper T cells (naïve, central memory, effector memory, and effector) were significantly lower in stage IV IBC patients, whereas reductions within CD8^+^ cytotoxic T lymphocytes were more concentrated in the memory subsets. CD4^+^ T lymphocytes enhance tumor antigen-specific immune responses by producing cytokines, and CD8^+^ T cells provide key adaptive anti-tumor immunity through their production of IFN-γ and cytolytic activity [30]. While naïve T cells have not yet been activated and exist in a resting state, effector T cells are short-lived and exhibit low proliferative capacity, but elicit potent functional responses toward an antigenic target [31]. The memory subsets provide more rapid and robust secondary responses upon re-exposure to antigens, whereupon they differentiate to an effector state. Central memory T cells have the most potent proliferative capability and longevity, but weakest functional responsiveness, while effector memory cells have intermediate properties between central memory and effector T cells [31].

It should be noted that lymphopenia is more common in patients with a variety of advanced tumors, as compared to those with localized disease [32]. In particular, reductions in peripheral CD4^+^ T lymphocytes are commonly observed in advanced cases of pancreatic cancer, melanoma, non-Hodgkin lymphoma, sarcoma, hepatocellular carcinoma, and breast cancer [32]. Lymphopenia, particularly the low frequency of CD4^+^ T cells, in patients with advanced cancer has also been shown to correlate with performance status, unfavorable prognostic factors, and worse survival [32, 33].

Our data support the findings of Reuben and Lee, which reported that the CD4/CD8 ratio of non-metastatic IBC patients was significantly higher than in metastatic IBC patients, because the non-metastatic IBC patients had a significantly greater reduction in CD4^+^ helper T lymphocytes than healthy controls [28]. In contrast, Mego et al. did not observe any alterations in CD4/CD8 T cell ratio between patients with metastatic or non-metastatic IBC and healthy normal donors [29]. Of note, the percentage of CD4^+^ T cells that were FoxP3^+^ and CD25^high^ (regulatory T cells; Treg) did not differ between our IBC patient and healthy control cohorts (data not shown), in contrast to the Mego et al. report of decreased numbers of Tregs in metastatic IBC patients compared to non-metastatic IBC patients and healthy controls [28].

While our study showed reduced numbers of peripheral NK cells in metastatic IBC, Reuben and Lee found no significant reductions in NK cells of metastatic or non-metastatic IBC patients, as compared to healthy controls [28]. NK cells constitute approximately 5% of the lymphocytes in healthy human peripheral blood and are involved in controlling tumor progression and metastases in a variety of contexts [34]. Two major NK cell subsets are found in human subjects that can be distinguished by their levels of CD56 expression, namely CD56^dim^ and CD56^bright^ [35]. CD56^bright^ NK cells make up approximately 2–10% of total NK cells in peripheral blood, are less mature, more apt to leave the vasculature, more efficient at producing cytokines, and less cytolytic than CD56^dim^ cells. The predominant cytolytic targets of NK cells are rare cells that have downregulated expression of class I MHC (MHC-I), which is normally expressed on healthy nucleated cells of the body [36]. MHC-I loss is a common mechanism by which tumors and virus-infected cells can evade recognition by cytolytic T cells, and NK cells can thereby overcome this potential immunologic evasion mechanism [27]. A counterbalance of signals from activating and MHC-I-binding inhibitory receptors on NK cells regulate their responsiveness [37]. Non-IBC tumors commonly express ligands for the NK cell activating receptors, DNAM-1 and NKG2D, which can increase their susceptibility to attack [38]. CD56^dim^ NK cells express the activating receptor CD16 (low-affinity FcγRIIIA) and mediate cytotoxicity by the directed exocytosis of perforins and granzymes from cytolytic granules, which perforate the target cell plasma cell membrane and trigger apoptosis, respectively [39]. A previous report described increased frequencies of immature and non-cytolytic NK cells in advanced non-IBC patients [40], although such a shift was not evident in our study. Instead, our data showed that IBC patients had higher expression levels of CD16, granzyme B, and perforin on their CD56^bright^ NK cells, suggesting that these immature cells are undergoing accelerated maturation to replenish the diminished mature CD56^dim^ population. In contrast to our observed increase in CD16 expression in stage IV IBC, a previous report showed decreased expression of activating NK cell receptors, including CD16, during progression of non-IBC, while inhibitory receptors increased and this correlated with decreased NK cell function, at least partially due to TGF-β1 in the TME [41].

Our study also provides further evidence that the immune system in some metastatic IBC patients has responded to the tumor at some stage of cancer development. We showed that IBC tumors from a subset of stage IV patients expressed moderate to high levels of PD-1 (18.2% of patients) and PD-L1 (36.4% of patients) on infiltrating immune cells by IHC analysis. In addition, we found a positive correlation between PD-L1 expression and PD-1 expression in our IBC tumor biopsies. Our results are consistent with Bertucci et al., who reported overexpression of PD-L1 mRNA in 38% of IBC patient tumors that were associated with increased B and CD8^+^ T cell gene expression signatures [18]. Similarly, two groups recently found that expression of PD-L1 in IBC tumor samples correlated with higher stromal tumor-infiltrating lymphocytes (sTIL) that were highly enriched in CD20^+^ B cells, and their combined presence was associated with better response to neoadjuvant therapy [17, 22]. We have expanded upon these results by further showing that expression of PD-1 and PD-L1 in our stage IV IBC samples correlated significantly with infiltration of CD20^+^ B cells and a trending correlation was noted for infiltration of CD3^+^ T cells. The invasion of the tumor stroma by sTIL is often associated with a better prognosis in ER-negative non-IBC [42]. Although breast cancer is considered moderately immunogenic, the presence of neoantigens seems to elicit an immune response, and infiltrating immune cells play an essential role in the host-defense mechanism against ER-negative non-IBC in both adjuvant and neoadjuvant studies [43, 44]. Van Berckelaer et al. recently showed that PD-L1 expression on sTIL was more frequently observed in IBC than non-IBC except in the Her2^+^ subtype [22]. PD-L1 expression on immune cells was seen in 38.6–42.9% of the IBC patients, and it was significantly higher than in non-IBC patients [22]. Hamm et al. also showed that some IBC tumors had high infiltration of CD8^+^ cells expressing PD-L1, and these had genetic profiles predictive of greater incidence of potential neoantigens [21].

As an extension of the previous studies, we also found correlations between PD-L1 expression in the stage IV IBC tumors and immune parameters in peripheral blood. Our results showed that the expression levels of PD-L1 in tumor tissues correlated positively with expression levels of cytolytic granule components (perforin and granzyme B) in peripheral blood CD4^+^ T and CD56^dim^ NK cells. The higher levels of these granule components are indicative of a previously activated state and consistent with the higher levels in immature CD56^bright^ NK cells, as compared to healthy controls. Furthermore, we found that higher expression of PD-L1 in the tumors also correlated with a shift from reduced percentages of naïve to increased frequency of effector memory CD8^+^ T cells in peripheral blood. Taken together, these results suggest that the CD4^+^ T and NK cells have been activated and effector memory CD8^+^ T cells have at some point expanded in response to expression of the immunosuppressive ligand in the tumor in a subset of the metastatic IBC patients. Despite these activation events, these immune cells have declined in overall numbers and presumably progressed to the classical exhausted state after chronic exposure to tumor. T cell exhaustion is a phenotype defined by poor effector function, such as reduced secretion of IL-2, IFN-γ, and TNF-α [45]. Reuben and Lee have further found that CD8^+^ T cells from the blood of patients with non-metastatic IBC had enhanced IFN-γ production responses upon T cell receptor (TCR)-stimulation, while IFN-γ production declined again toward levels of healthy donors in patients with metastatic IBC [28]. These results suggest that CD8^+^ T cell responsiveness may be enhanced in non-metastatic IBC patients. All of these observations imply that immunotherapy should be considered as a potential treatment for those patients exhibiting increased expression of PD-1 and/or PD-L1 in tumor and/or increased numbers of cytolytic effector memory T cells in peripheral blood. In such patients, blockade of the PD-1/PD-L1 inhibitory axis has the potential to reactivate antigen-experienced, exhausted T cells toward the tumor and thereby might improve clinical outcome. In fact, four clinical trials are currently testing the efficacy of PD-1 or PD-L1 blockade in IBC patients (NCT03515798, NCT02411656, NCT03742986, and NCT03202316).

The data reported in our study support the concept of progressive immune dysfunction as IBC advances to highly metastatic clinical behavior. In fact, IBC is associated with early metastatic dissemination as suggested by higher numbers of circulating tumor cells (CTCs) compared to other forms of breast cancer. It has been shown that IBC patients with higher numbers of CTCs also have a more compromised immune status, which is characterized by reduced percentages of CD4^+^ helper T cells, higher percentage of Treg cells, and reduced cytokine-producing CD8^+^ T cells [29]. Peripheral blood immune cells can contribute to an unfavorable environment for CTC survival, since innate and adaptive immune mechanisms are purportedly responsible for controlling tumor dissemination and, perturbations in the immune surveillance could favor an environment conducive for the survival and dissemination of CTCs, ultimately leading to cancer progression [46]. We can hypothesize that the lower level of immune surveillance in lymphopenic metastatic IBC patients could facilitate additional metastasis, but the ultimate causes of immune dysfunction in IBC need to be better defined. To improve mechanistic understanding, Reddy et al. recently showed greater macrophage infiltration in IBC tumors than in other breast cancers [47], and they further showed that IBC tumors with increased infiltration of mast cells were associated with poorer clinical responses to neoadjuvant chemotherapy [48]. Also, Valeta-Magara et al. recently showed that IBC tumors produce chemokines and cytokines that recruit monocytes and polarize macrophages to the M2 phenotype, which are immunosuppressive and tumor-promoting [49]. Thus, the accumulating evidence suggests that M2 macrophages and the PD-1/PD-L1 axis contribute to the immunosuppressive TME in IBC, although more studies are clearly needed.

## Conclusions

Through this work, we investigated immune parameters in peripheral blood of metastatic IBC patients by using flow cytometry-based immune phenotyping, which is different from most of the previous studies in IBC that focused on infiltrating immune cells. We further compared these parameters with PD-1 and PD-L1 expression and T and B cell infiltration in IBC tumor biopsies by IHC. A limitation of our work is the small cohort of patients that were analyzed. Nonetheless, IBC is a rare cancer in need of more detailed study, and we provide comparison between immune parameters in peripheral blood and within the TME.

Our study provides evidence that the immune system of a subset of patients with stage IV IBC has responded to the tumor. IBC tumor biopsies from most patients expressed clearly detectable levels of PD-1 (18.2% of patients) and PD-L1 (36.4% of patients), as defined by staining scores of 3–4 (moderate to high), and expression levels of this checkpoint receptor/ligand pair were correlated in the TME. Interestingly, increased PD-L1 expression in tumor also correlated with higher cytolytic granule components in NK and CD4^+^ helper T cells from blood, as well as greater frequency of effector memory and lower percentage of naïve CD8^+^ cytotoxic T cells in the blood. PD-1 expression in tumor also correlated with increased CD20^+^ B cell TIL. These results provide rationale to consider PD-1/PD-L1 blocking immunotherapy as a potential treatment for IBC patients exhibiting increased expression of PD-1 and/or PD-L1 in tumor. In such patients, blockade of the PD-1/PD-L1 inhibitory axis has the potential to reactivate antigen-experienced exhausted T cells toward the tumor and thereby might improve clinical outcome.

## Supplementary Information

**Additional file 1.**

## Data Availability

The data of this work will be made available upon reasonable request.

## Abbreviations

CD8^+^ T: Cytotoxic T lymphocytes
CD4^+^ T: Helper T lymphocytes
CTC: Circulating tumor cell
Her2 or ErbB2: Receptor tyrosine-protein kinase erbB-2
ER or ESR1: Estrogen receptor alpha
FDR: False Discovery Rate
FFPE: Formalin-fixed paraffin-embedded
IBC: Inflammatory breast cancer
IHC: Immunohistochemistry
NK: Natural killer
PD-1: Programmed cell death protein 1
PD-L1: Programmed death-ligand 1
PR: Progesterone receptor
OS: Overall survival
PR: Progesterone receptor
sTIL: Stromal tumor-infiltrating lymphocytes
TCR: T cell receptor
TN: Triple-negative breast cancer
Treg: Regulatory T cells
TIL: Tumor-infiltrating lymphocytes
TME: Tumor microenvironment
