# Supplementary material for: Immune phenotype of patients with stage IV metastatic inflammatory breast cancer
Source: Breast Cancer Res. 2020 Dec 2;22:134. doi: 10.1186/s13058-020-01371-x (PMC7709446; doi:10.1186/s13058-020-01371-x)
Supplement: Supplementary file 1 — Additional file 1. [file 13058_2020_1371_MOESM1_ESM.pdf]

Fluorophore	NK	Monocyte	T	B	Treg (Fix/Perm)	Perforin/ Granzyme (Fix/Perm)
FITC or Alexa Fluor 488	**CD56** NCAM16.2 (BD)	**PD-1** MIH4 (EB)	**PD-1** MIH4 (EB)	**Lambda** MHL-38 (BL)	**CD25** 2A3 (BD)	**Perforin** dG9 (BL)
PE	**NKp44** P44-8 (BL)	**CD14** HCD14 (BL)	**CD62L** DREG-56 (BL)	**Kappa** MHK-49 (BL)		**Granzyme B** GB11 (BL)
APC	**NKG2D** 1D11 (BL)	**CD56** NCAM16.2 (BD)	**CD56** NCAM16.2 (BD)	**CD5** UCHT2 (BL)	**FoxP3** 206D (EB)	**CD56** NCAM16.2 (BD)
PerCP/Cy5.5	**CD45** 2D1 (BD)	**CD45** 2D1 (BD)	**CD45** 2D1 (BD)	**CD45** 2D1 (BD)	**CD45** 2D1 (BD)	**CD45** 2D1 (BD)
Cy7/APC or APCH7	**CD3** SK7 (BD)	**CD3** SK7 (BD)	**CD3** SK7 (BD)	**CD10** HI10a (BL)	**CD3** SK7 (BD)	**CD3** SK7 (BD)
AlexaFluor 700		**CD16** 3G8 (BL)	**CD8** HIT8a (BL)	**CD27** O323 (BL)	**CD8** HIT8a (BL)	**CD8** HIT8a (BL)
Cy7/PE		**CD19** HIB19 (EB)	**CD45RA** L48 (BD)	**CD19** HIB19 (EB)		
PerCP	**Propidium Iodide** 100 ng/ml	**Propidium Iodide** 100 ng/ml	**Propidium Iodide** 100 ng/ml	**Propidium Iodide** 100 ng/ml		
Pacific Blue	**CD69** FN50 (BL)	**CD69** FN50 (BL)	**CD4** OKT4 (BL)	**CD20** L27 (BD)	**CD4** OKT4 (BL)	**CD4** OKT4 (BL)

Supplementary Table S1 - Antibody Staining Panel for Flow Cytometry. Each column represents a pool of antibodies used to stain an aliquot of PBMC with the cell type focus listed in the header. Rows designate the fluorophore label of individual antibodies as listed in the first column. The target antigens are listed in bold, followed by hybridoma clone and source (BD = BD Biosciences, San Jose, CA; EB = eBiosciences/Affymetrix, San Diego, CA; BL = Biolegend, San Diego, CA).

Myeloid Cell Parameters	Lymphocyte Counts	T cell Parameters
Classical Monocyte %	Total Lymphocytes per µl	CD56+ % of T
Intermediate Monocyte %	CD3+ T cells per µl	CD4+ Central Memory
Non-Classical Monocytes %	CD8+ T cells per µl	CD4+ Effector
Classical Monocyte PD-1 GMFI	CD4+ cells per µl	CD4+ Effector Memory
Intermediate Monocyte PD-1 GMFI	CD4+ CD8+ cells per µl	CD4+ Naïve
Non-Classical Monocytes PD-1 GMFI	NK cells per µl	CD8+ Central Memory
Classical Monocyte CD69 GMFI	B cells per µl	CD8+ Effector
Intermediate Monocyte CD69 GMFI	CD4/CD8 ratio	CD8+ Effector Memory
Non-Classical Monocyte CD69 GMFI	CD4+ Central Memory Count	CD8+ Naïve
Granulocytes	CD4+ Effector Count	NKG2D %+
Granulocytes PD1 GMFI	CD4+ Effector Memory Count	NKG2D+ GMFI
Granulocytes CD69 GMFI	CD4+ Naïve Count	PD-1 GMFI
**NK Cell Parameters**	CD8+ Central Memory Count	CD4+ PD-1 GMFI
% $CD56^{bright}$	CD8+ Effector Count	CD8+ PD-1 GMFI
$CD56^{dim}$ NKG2D GMFI	CD8+ Effector Memory Count	CD56+ T PD-1 GMFI
$CD56^{bright}$ NKG2D GMFI	CD8+ Naïve Count	CD4+ Central Memory PD-1 GMFI
$CD56^{dim}$ NKp44 % +	**B Cell Parameters**	CD4+ Effector PD-1 GMFI
$CD56^{bright}$ NKp44 %+	CD20+ CD10+ Immature B cells	CD4+ Naïve PD-1 GMFI
$CD56^{dim}$ CD69 GMFI	CD20+ CD27- Naïve B cells	CD8+ Central memory PD-1 GMFI
$CD56^{bright}$ CD69 GMFI	CD20+ CD27+ Memory B cells	CD8+ Effector PD-1 GMFI
$CD56^{dim}$ Perforin GMFI	CD20- CD27+ Plasma cells	CD8+ Effector Memory PD-1 GMFI
$CD56^{bright}$ Perforin GMFI		CD8+ Naïve PD-1 GMFI
$CD56^{dim}$ Granzyme B GMFI		CD4+ Treg (FoxP3+)
$CD56^{bright}$ Granzyme B GMFI		
$CD56^{dim}$ PD-1 GMFI		
$CD56^{bright}$ PD-1 GMFI		
$CD56^{dim}$ CD16 GMFI		
$CD56^{bright}$ CD16 GMFI		
$CD56^{dim}$ NKp44 GMFI		
$CD56^{bright}$ NKp44 GMFI		

Supplementary Table S2. Specific immune parameters analyzed in peripheral blood by flow cytometry.

Patient #	Sample collection	Blood collection	Time after blood	PD-1 score	PD-L1 score	Description	Pathology report
2	Month 22	Month 12	10 months	0	0	skin, punch biopsy, left breast	carcinoma infiltrating dermis and dermal lymphatics consistent with breast primary
2	Month 12	Month 12	same day*	0	0	Skin, right scapula, punch biopsy	Poorly differentiated adenocarcinoma in dermal lymphatics
3	Month 34	Month 20	15 months	1	0	Right inframammary crease, punch biopsy	Dermis and dermal lymphatics with high grade carcinoma, consistent with breast origin
6	Month 8	Month 8	same day	1	2	skin, right upper chest, punch biopsy	carcinoma in dermis
7	Month 21	Month 21	8 days	0	0	skin, left posterior shoulder, medial	High grade carcinoma in dermis and dermal lymphatics
9	Month 4	Month 4	1 day	3	3	skin, right lateral breast, punch biopsy	Ductal carcinoma within dermal lymphatics and invading dermis
9	Month 4	Month 4	20 days	1	4	4R lymph nodes, excision	metastatic carcinoma in 6/6 lymph nodes
10	Month 32	Month 32	same day	1	2	skin, left chest wall, punch biopsy	carcinoma within dermal lymphatics, consistent with the patient's history of ductal carcinoma
11	Month 17	Month 13	4 months	3	4	skin, right chest wall, punch biopsy	Carcinoma with dermal lymphatics consistent with breast primary (ductal carcinoma)
11	Month 20	Month 13	5 months	2	4	tumor- right chest wall, punch biopsy	high grade carcinoma in dermal lymphatics consistent with breast primary (ductal carcinoma)
13	Month 1	Month 1	3 days	0	1	left breast core biopsy	invasive duct carcinoma grade III
13	Month 1	Month 1	3 days	2	2	left axillary mass, biopsy	Invasive ductal carcinoma grade III
14	Month 20	Month 13	7 months	0	4	liver, core needle biopsy	Metastatic carcinoma consistent with breast origin ER-PR-
15	Month 22	Month 22	4 weeks	0	1	left breast mass, core biopsy	Invasive ductal carcinoma, nuclear grade 3
16	Month 27	Month 26	3 days	2	4	skin, right breast, punch biopsy	High grade ductal carcinoma, involves the entire dermis
16	Month 32	Month 26	5 months	1	2	skin, right breast in area of mastectomy, punch biopsy	High grade ductal carcinoma, involves the dermis, with lymphatic invasion

*yellow highlighting designates IHC samples obtained within one month of blood draw

Supplementary Table S3. Pathological characteristics of 16 tumor samples from 11 of the metastatic IBC patients analyzed for PD-1 and PD-L1 by immunohistochemistry. Yellow highlights are IHC samples obtained within one month of blood that were used for correlation analysis shown in Figure 5.
